# Supplementary material for: Structural basis of HIV-1 Vif-mediated E3 ligase targeting of host APOBEC3H
Source: Nat Commun. 2023 Aug 28;14:5241. doi: 10.1038/s41467-023-40955-x (PMC10462622; doi:10.1038/s41467-023-40955-x)

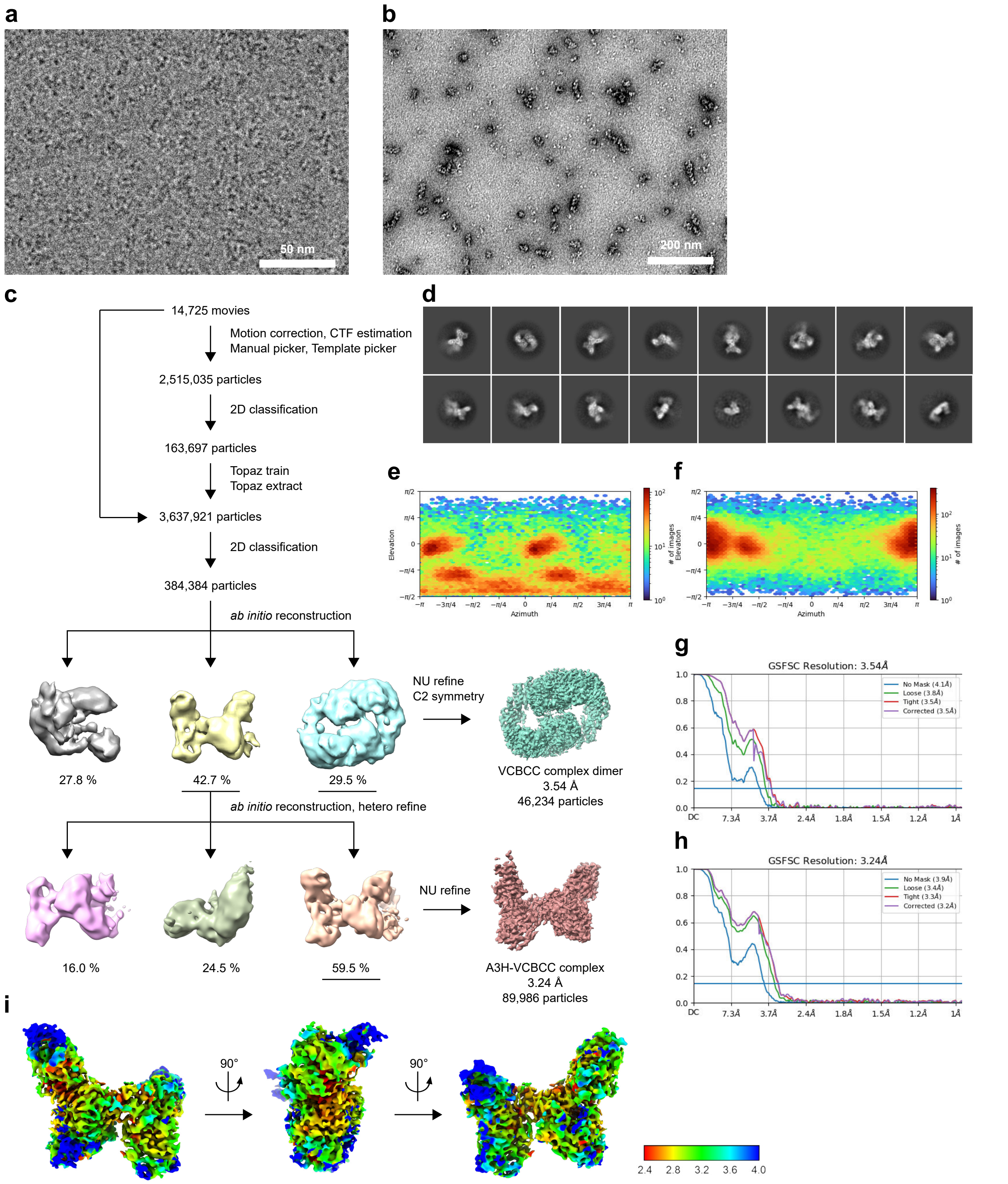

Supplementary Fig. 1. Workflow and intermediate results of electron microscopy imaging, image processing, 3D reconstruction, and resolution evaluation of the A3H-VCBCC complex and the VCBCC complex dimer. a, Representative cryo-EM raw image. **b,** Representative negative-stain EM raw image. **c,** Cryo-EM image processing workflow. **d,** Representative 2D class averages. **e-f,** Angular distribution plot of the particles containing (**e**) the VCBCC complex dimer and (**f**) the A3H-VCBCC complex. **g-h,** Global resolution estimation of (**g**) the VCBCC complex dimer and (**h**) the A3H-VCBCC complex based on the gold standard Fourier shell correlation (FSC) coefficient of 0.143 criterion. **i,** Local resolution evaluation of the A3H-VCBCC complex.

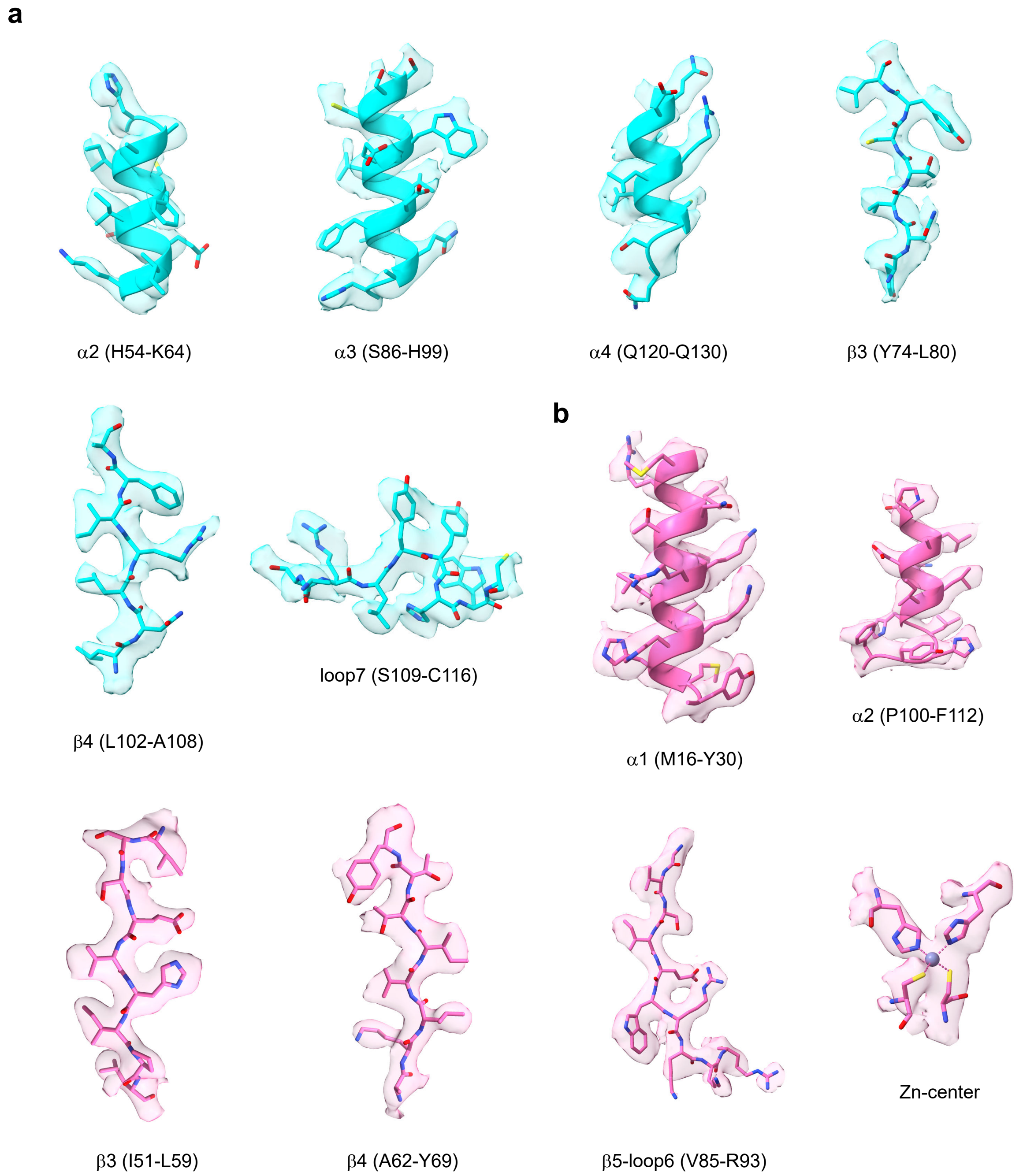

Supplementary Fig. 2. Regions of the cryo-EM density maps superposed with atomic models of the components of A3H-VCBCC complex. Segmented cryo-EM densities of representative local regions of A3H (**a**) and Vif (**b**) are shown as semi-transparent surfaces superposed with atomic models of amino acid side chains (ribbons and sticks).

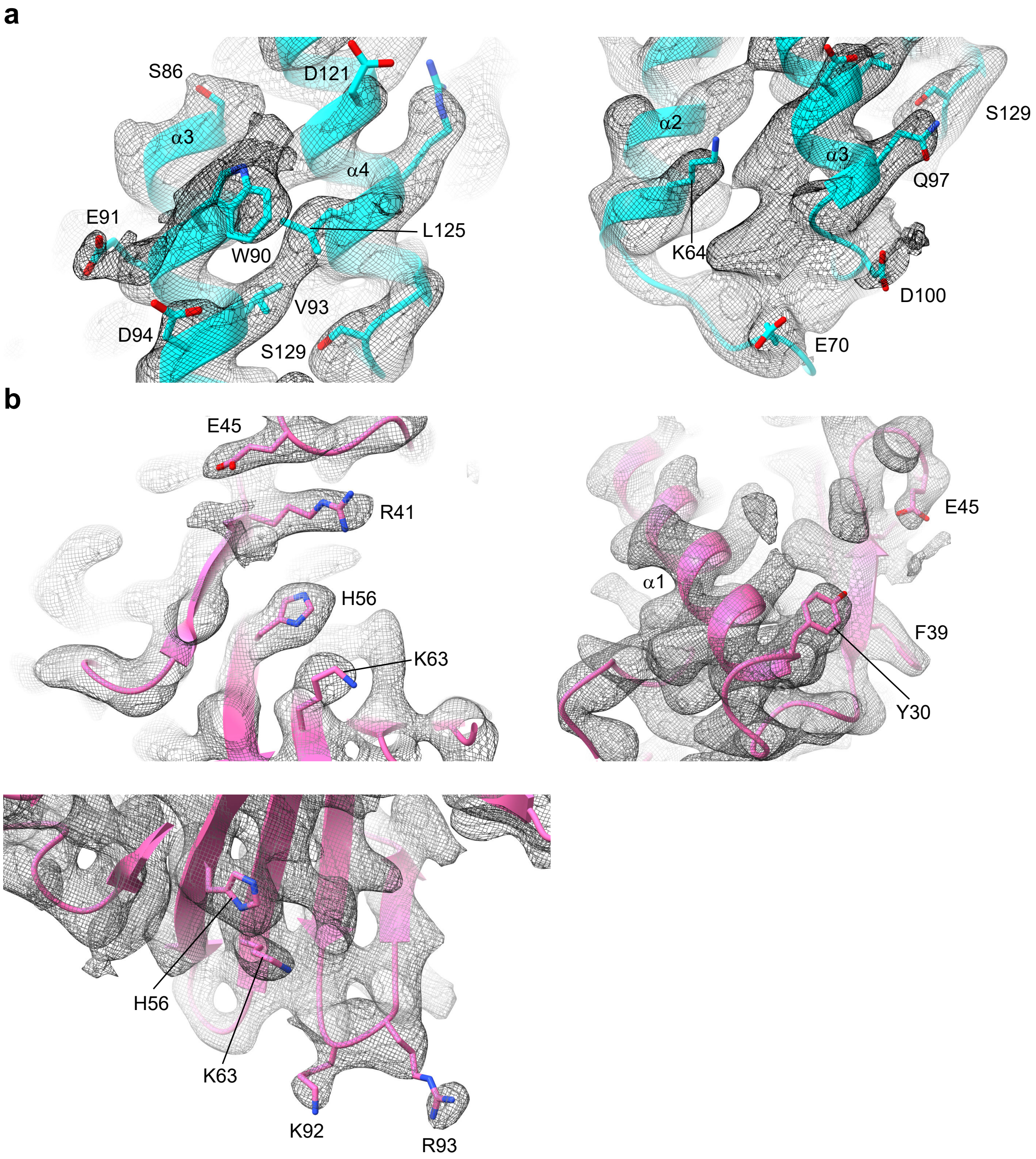

Supplementary Fig. 3. Cryo-EM densities of the key interface residues involved in the A3H-Vif interactions. (**a**) Densities superimposed to the A3H model, highlighting the Vif-interface residues. (**b**) Densities superimposed to the Vif model, highlighting the A3H-interface residues.

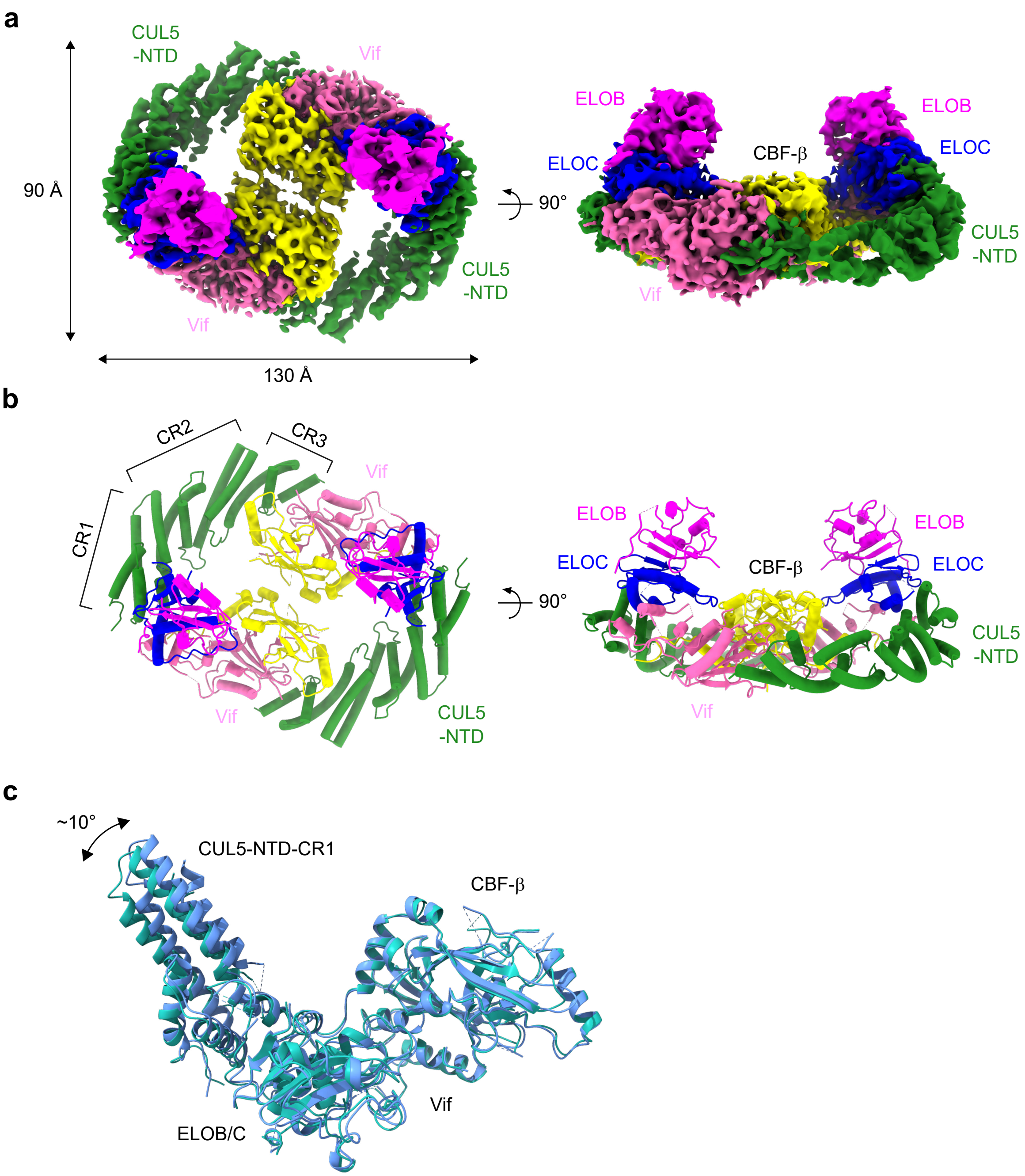

Supplementary Fig. 4. Structure of the VCBCC complex dimer. Two orthogonal views of (**a**) the cryo-EM density map and (**b**) the resultant atomic model of the VCBCC complex dimer. **c,** Superimposition of VCBCC subregion in A3H-VCBCC complex (light sea green) and VCBCC dimer (cornflower blue). For CUL5, only the CR1 of CUL5-NTD (aa:12-151) is displayed for comparison.

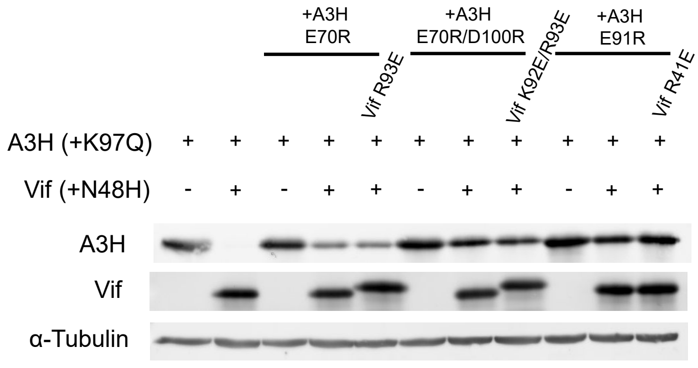

Supplementary Fig. 5. Vif-mediated degradation assay of charge-swapped mutants. The mutations on the A3H and Vif residues responsible for electrostatic interactions (Figs. 3g and h) were combined to see whether the degradation of A3H can be restored. The result showed no detectible restoration of the degradation, indicating that simple charge swapping of the amino acid side chains is insufficient to recover electrostatic bonds between A3H and Vif.

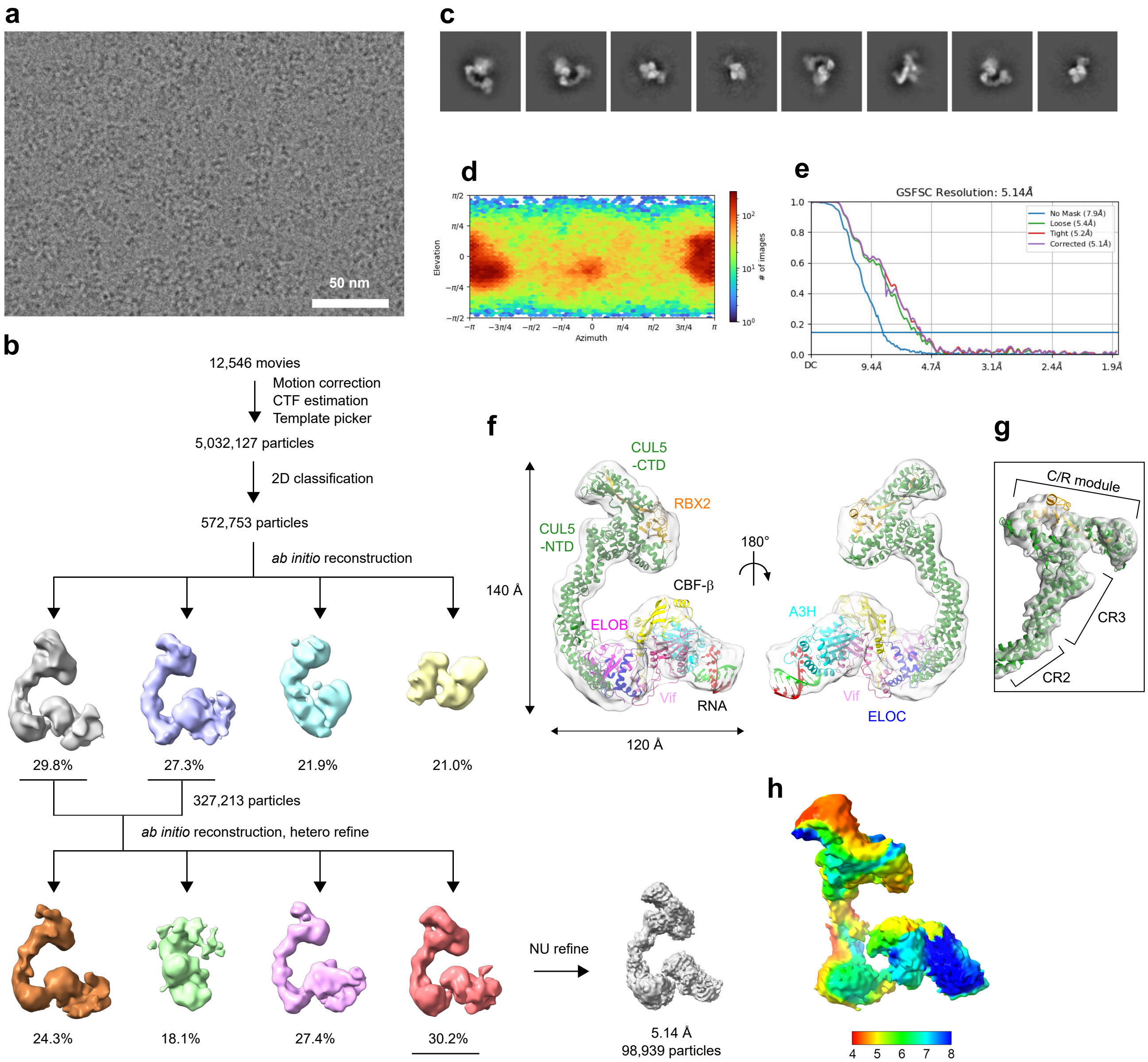

Supplementary Fig. 6. Workflow and intermediate results of electron microscopy imaging, image processing, 3D reconstruction, and resolution evaluation of the A3H-VCBCCR complex. a, Representative cryo-EM raw image. Scale bars: 20 nm. **b,** Cryo-EM image processing workflow. **c,** Representative 2D class averages. **d,** Angular distribution plot of the particles containing the A3H-VCBCCR complex. **e,** Global resolution estimation of the A3H-VCBCCR complex based on the gold standard Fourier shell correlation (FSC) coefficient of 0.143 criterion. **f,** Atomic model fitting of A3H-VCBCCR complex. Atomic models of A3H-VCBC subcomplex from this study and CUL5/RBX2 complex (PDBID: 6V9I) were docked into the cryo-EM density (semi-transparent gray). **g,** Close-up view of the superimposition of atomic model into the A3H-VCBCCR cryo-EM density around the region of CUL5 CR2-CR3 and the C/R module. **h**, Local resolution evaluation of the A3H-VCBCCR complex.

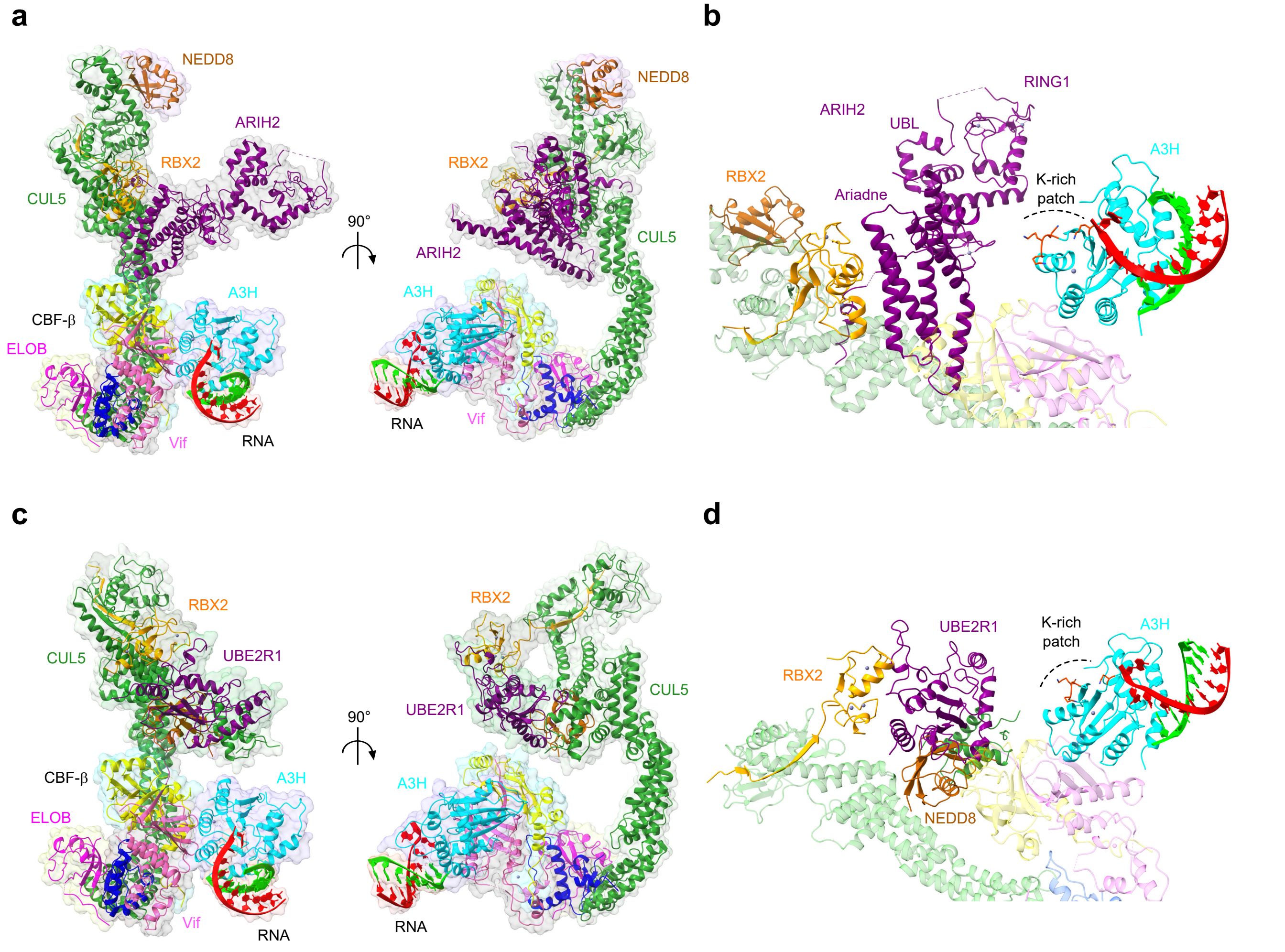

Supplementary Fig. 7. Extended models of A3H-Vif-Cul5 E3 ligase for ubiquitin transfer. a-b, ARIH2-bound model (PDB ID: 7ONI) of the A3H-Vif-CUL5 E3 ligase complex for mono-ubiquitination of A3H. **c-d,** UBE2R1-bound model (PDB ID: 6NYO, 6TTU) of the A3H-Vif-CUL5 E3 ligase complex for poly-ubiquitination of A3H.

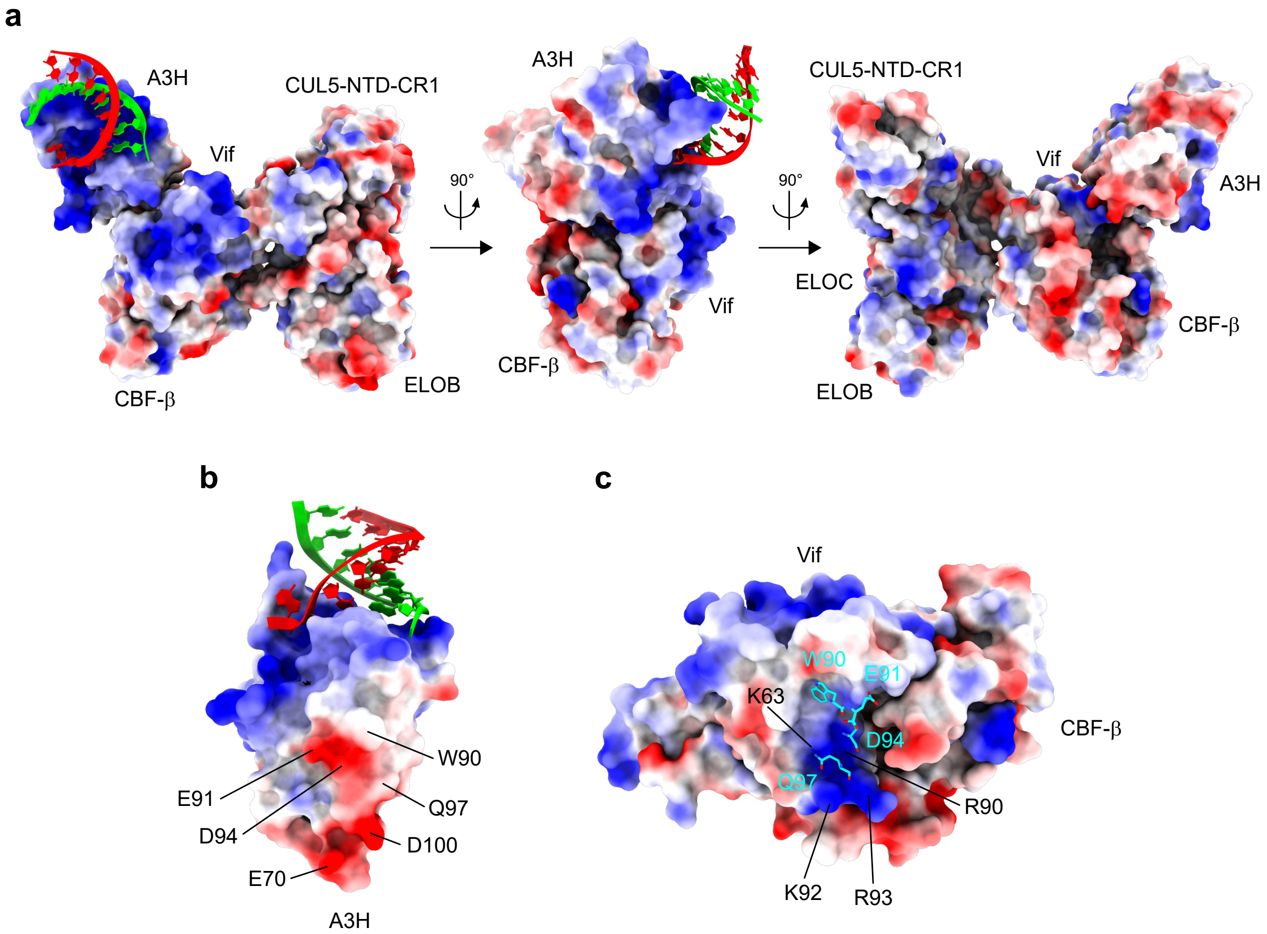

Supplementary Fig. 8. Surface electrostatic potential of A3H-VCBCC complex. a, Three orthorhombic views of the electrostatic potential of the A3H-VCBCC complex. **b,** Electrostatic potential of the Vif-binding surface of A3H. **c,** Electrostatic potential of the A3H-binding surface of Vif/CBF-β. Vif residues K63/K92/R93 create highly positively charged surface. Key Vif-binding residues of A3H are shown in sticks. Vif K63 is in contact with the sensitivity-modulating residue Q97 of A3H. The surface area is colored according to the calculated electrostatic potential from -10.0 kT/e (red) to +10.0 kT/e (blue).

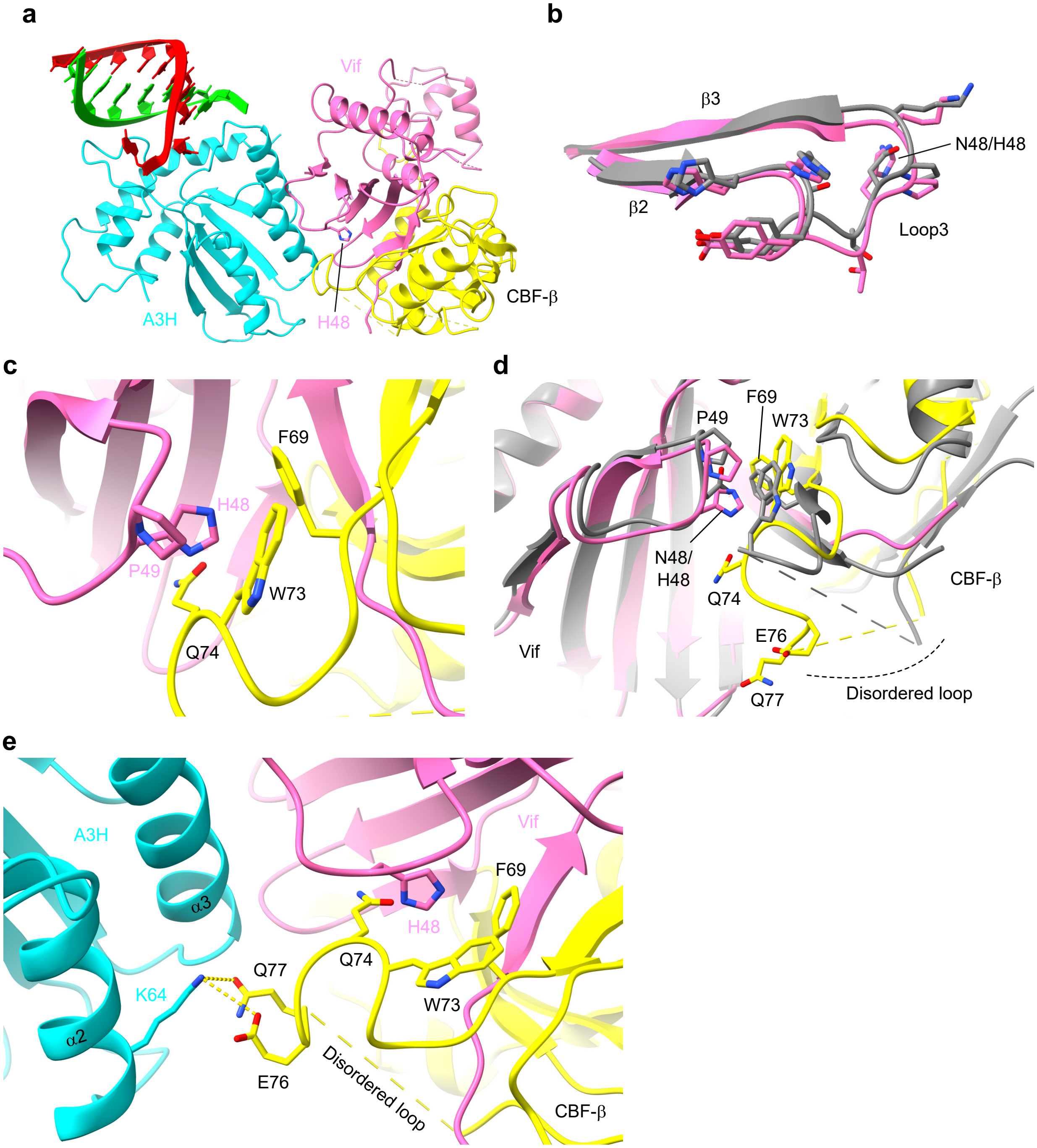

Supplementary Fig. 9. Details of A3H-Vif interactions involving Vif polymorphic residue at position 48. a, Location of Vif H48 in the A3H-VCBCC complex structure showing that the histidine is not directly engaged in binding to A3H. **b,** Superimposition of β2-loop3-β3 region of Vif in the A3H-VCBCC complex (pink) and Vif in the crystal structure of VCBCC complex (PDBID: 4N9F, dark grey). **c,** Vif and CBF-β residues around the Vif H48. The H48 packs with CBF-β F69/W73 as well as Vif P49. **d,** Superimposition of Vif-CBF-β subregion of the A3H-VCBCC complex (pink and yellow) and the same subregion of the crystal structure of VCBCC complex (dark grey). **e,** The interaction between Vif H48 and CBF-β F69/W73 enables the CBF-β E76/Q77 on the flexible loop to interact with A3H K64, thus stabilizing the A3H-VCBCC complex.

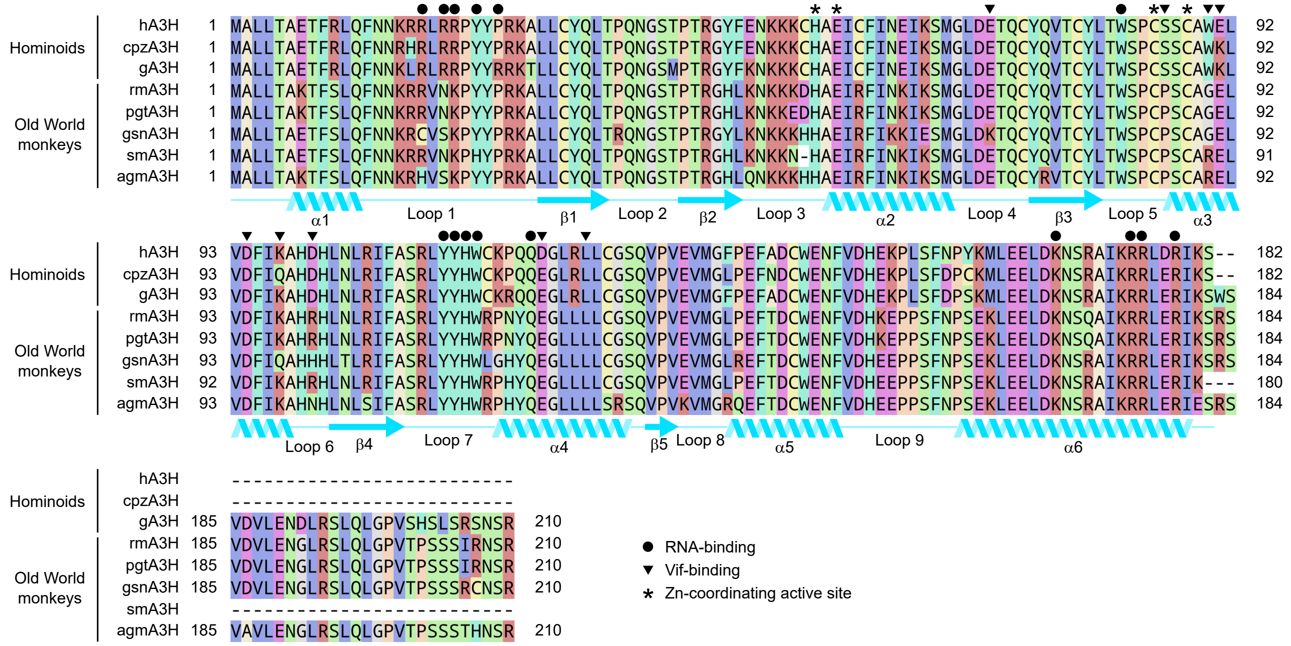

Supplementary Fig. 10. Sequence alignment of primate A3H. The functionally important residues in human A3H are marked for comparison with other primate A3Hs. Amino acid residues responsible for RNA-binding, Vif-binding, and the zinc-coordinating cytidine deaminase active site are marked with circles, triangles, and asterisks, respectively. hA3H, human; cpzA3H, chimpanzee; gA3H, gorilla; rmA3H, rhesus macaque; pgtA3H, pig-tailed macaque; gsnA3H, golden snub-nosed monkey; smA3H, sooty mangabey; agmA3G, African green monkey.

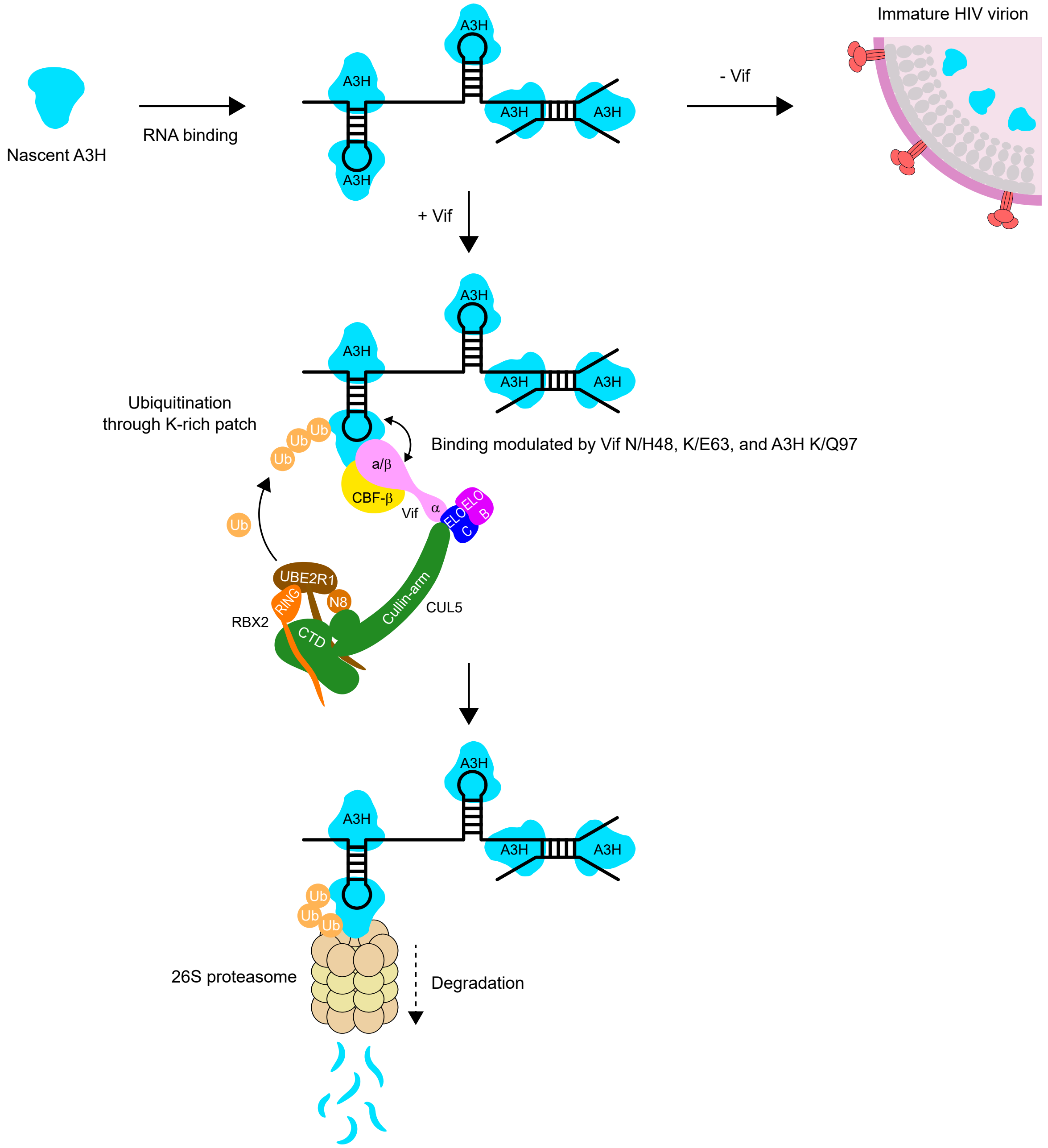

Supplementary Fig. 11. Model of Vif-mediated antagonism of A3H. In HIV-1ΔVif infection, newly synthesized A3H first binds to viral genomic RNA or host cellular RNA through recognizing structured regions of RNA. A3H then dimerizes and assembles into a high molecular mass ribonucleoprotein complex, which can be packaged into budding HIV virions for restricting the subsequent viral replication. When Vif is present in HIV-1, Vif redirects the CUL5 E3 ligase complex to target A3H for ubiquitination. The binding of Vif to A3H is modulated by polymorphic residues at position 48 and 63 of Vif, and 97 of A3H. Vif-CUL5 E3 ligase complex ubiquitinates A3H through K-rich patch around loops 1 and 3 of A3H. Poly-ubiquitinated A3H undergoes proteolytic degradation in 26S proteasome, thus HIV-1 overcomes the innate immunity by A3H. Schematics adapted from Ito, F. *et al*. Structural basis for HIV-1 antagonism of host APOBEC3G via Cullin E3 ligase. Sci Adv 9, eade3168, doi:10.1126/sciadv.ade3168 (2023).

Supplementary Table 1. Cryo-EM data collection, refinement, and validation statistics

	A3H-VCBCC complex (PDB: 8FVI) (EMDB: EMD-29488)	VCBCC dimer (PDB: 8FVJ) (EMDB: EMD-29489)	A3H-VCBCCR complex (EMDB: EMD-29490)
**Data collection**			
Magnification	165,000	165,000	150,000
Voltage (kV)	300	300	200
Electron exposure ($e^-/Å^2$)	50	50	40
Defocus range (μm)	-1.0 to -3.0	-1.0 to -3.0	-1.2 to -3.0
Pixel size (Å)	0.51	0.51	0.92
Symmetry imposed	C1	C2	C1
Initial particle images	3,637,921	3,637,921	5,076,706
Final particle images	89,986	46,234	49,608
Map resolution (Å)	3.24	3.54	5.14
FSC threshold	0.143	0.143	0.143
Map resolution range (Å)	2.4 - 4.6	2.9 - 5.8	4.0 - 10.0
**Refinement**			
Initial model used (PDB)	4N9F, 5W45	4N9F	
Model resolution (Å)	3.5	3.9	
FSC threshold	0.5	0.5	
Map sharpening B factor ($Å^2$)	-83.6	-81.3	
No. non-hydrogen atoms	6774	12562	
Protein residues	777	1536	
Nucleotides	19	0	
Ligands	2 Zn	2 Zn	
B-factors			
Protein	59.54	78.42	
Nucleotide	64.40	-	
Ligand	77.35	52.82	
R.m.s. deviations			
Bond lengths (Å)	0.003	0.003	
Bond angles (°)	0.572	0.656	
Validation			
MolProbity score	2.33	2.14	
Clash score	14.63	15.86	
Poor rotamers (%)	2.15	0.73	
Ramachandran plot			
Favored (%)	93.99	93.26	
Allowed (%)	6.01	6.74	
Disallowed (%)	0.00	0.00	

Uncropped western blot images shown in Supplementary Fig. 5

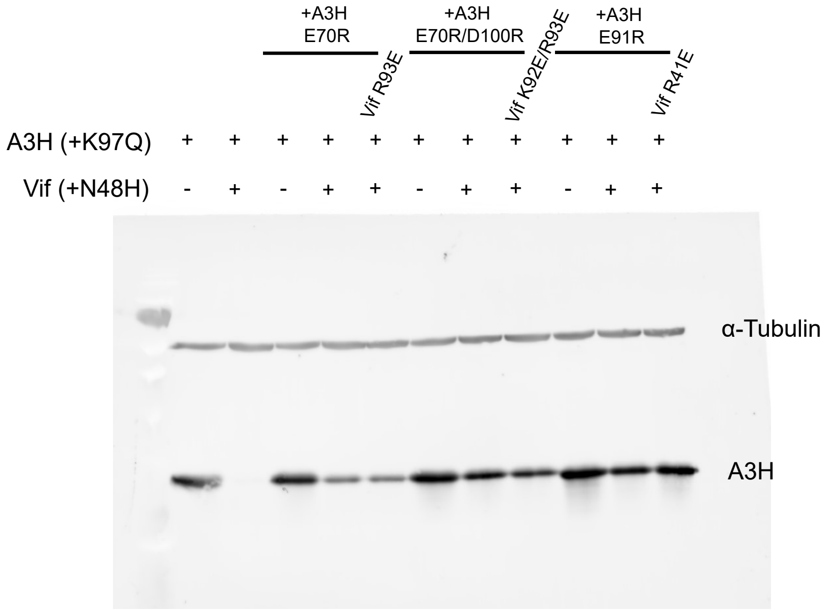

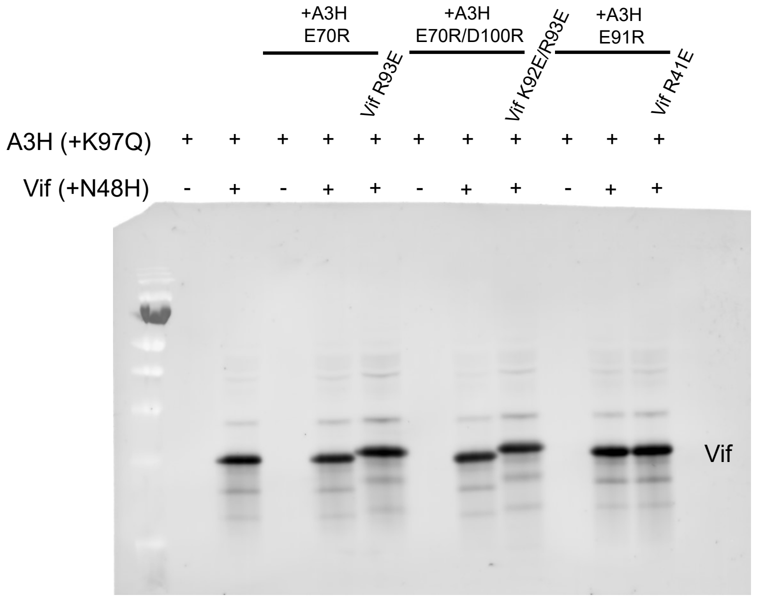

Supplement: Supplementary file 1 — Supplementary Information [file 41467_2023_40955_MOESM1_ESM.pdf]
