# Supplementary material for: Structural basis of HIV-1 Vif-mediated E3 ligase targeting of host APOBEC3H
Source: Nat Commun. 2023 Aug 28;14:5241. doi: 10.1038/s41467-023-40955-x (PMC10462622; doi:10.1038/s41467-023-40955-x)
Supplement: Supplementary file 3 — Description of Additional Supplementary Files [file 41467_2023_40955_MOESM3_ESM.pdf]

File name: Supplementary Movie 1
Description: Cryo-EM map of the A3H-VCBCC complex and its superimposition with the corresponding atomic model.

File name: Supplementary Movie 2
Description: Cryo-EM structure of the A3H-VCBCC complex highlighting the A3H-Vif interface around A3H α3 and α4.

File name: Supplementary Movie 3
Description: Surface electrostatic potential of the A3H-VCBCC complex highlighting the A3H-Vif interface.
