# Supplementary material for: Structural basis of HIV-1 Vif-mediated E3 ligase targeting of host APOBEC3H
Source: Nat Commun. 2023 Aug 28;14:5241. doi: 10.1038/s41467-023-40955-x (PMC10462622; doi:10.1038/s41467-023-40955-x)
Supplement: Supplementary file 7 — Reporting Summary [file 41467_2023_40955_MOESM7_ESM.pdf]

nature portfolio

Corresponding author(s): Xiaojiang Chen

Last updated by author(s): Jul 15, 2023

# Reporting Summary

Nature Portfolio wishes to improve the reproducibility of the work that we publish. This form provides structure for consistency and transparency in reporting. For further information on Nature Portfolio policies, see our Editorial Policies and the Editorial Policy Checklist.

## Statistics

For all statistical analyses, confirm that the following items are present in the figure legend, table legend, main text, or Methods section.

| n/a | Confirmed | |
|---|---|---|
| ☐ | ☒ | The exact sample size (*n*) for each experimental group/condition, given as a discrete number and unit of measurement |
| ☐ | ☒ | A statement on whether measurements were taken from distinct samples or whether the same sample was measured repeatedly |
| ☐ | ☒ | The statistical test(s) used AND whether they are one- or two-sided<br>*Only common tests should be described solely by name; describe more complex techniques in the Methods section.* |
| ☒ | ☐ | A description of all covariates tested |
| ☒ | ☐ | A description of any assumptions or corrections, such as tests of normality and adjustment for multiple comparisons |
| ☐ | ☒ | A full description of the statistical parameters including central tendency (e.g. means) or other basic estimates (e.g. regression coefficient) AND variation (e.g. standard deviation) or associated estimates of uncertainty (e.g. confidence intervals) |
| ☐ | ☒ | For null hypothesis testing, the test statistic (e.g. *F*, *t*, *r*) with confidence intervals, effect sizes, degrees of freedom and *P* value noted<br>*Give P values as exact values whenever suitable.* |
| ☒ | ☐ | For Bayesian analysis, information on the choice of priors and Markov chain Monte Carlo settings |
| ☒ | ☐ | For hierarchical and complex designs, identification of the appropriate level for tests and full reporting of outcomes |
| ☒ | ☐ | Estimates of effect sizes (e.g. Cohen's *d*, Pearson's *r*), indicating how they were calculated |

*Our web collection on statistics for biologists contains articles on many of the points above.*

## Software and code

Policy information about availability of computer code

| | |
|---|---|
| Data collection | EPU v3.0 (Thermo Fisher Scientific) |
| Data analysis | cryoSPARC v4.1.2, Phenix v1.20.1, Coot v0.9.8.2, UCSF Chimera v1.16, UCSF ChimeraX v1.5, Topaz v0.2 |

For manuscripts utilizing custom algorithms or software that are central to the research but not yet described in published literature, software must be made available to editors and reviewers. We strongly encourage code deposition in a community repository (e.g. GitHub). See the Nature Portfolio guidelines for submitting code & software for further information.

## Data

Policy information about availability of data

All manuscripts must include a data availability statement. This statement should provide the following information, where applicable:

- Accession codes, unique identifiers, or web links for publicly available datasets
- A description of any restrictions on data availability
- For clinical datasets or third party data, please ensure that the statement adheres to our policy

The atomic models have been deposited in the PDB with accession codes: 8FVI (A3H-VCBCC complex) and 8FVJ (VCBCC complex dimer). The cryo-EM maps have been deposited in the EMDB with accession codes: EMD-29488 (A3H-VCBCC complex), EMD-29489 (VCBCC complex dimer), and EMD-29490 (A3H-VCBCCR complex). Raw electron microscopy data files have been deposited in the Electron Microscopy Public Image Archive (EMPIAR) with accession code EMPIAR-11423 (A3H-VCBCCR complex) and EMPIAR-11424 (A3H-VCBCC complex and VCBCC complex dimer). Source data are provided with this paper.

## Human research participants

Policy information about studies involving human research participants and Sex and Gender in Research.

| | |
|---|---|
| Reporting on sex and gender | N/A |
| Population characteristics | N/A |
| Recruitment | N/A |
| Ethics oversight | N/A |

Note that full information on the approval of the study protocol must also be provided in the manuscript.

# Field-specific reporting

Please select the one below that is the best fit for your research. If you are not sure, read the appropriate sections before making your selection.

☒ Life sciences ☐ Behavioural & social sciences ☐ Ecological, evolutionary & environmental sciences

For a reference copy of the document with all sections, see nature.com/documents/nr-reporting-summary-flat.pdf

# Life sciences study design

All studies must disclose on these points even when the disclosure is negative.

| | |
|---|---|
| Sample size | No methods were used that would require a predetermined sample size due to the nature of the current study related to Vif-mediated degradation assay, in vitro ubiquitination assay, and single-particle cryo-EM. Vif-mediated degradation assay and in vitro ubiquitination assay were performed independently triplicates. The reproducibility of the triplicates made us confident that the sample size was sufficient. The sample size in cryo-EM was considered adequate since the addition of extra data did not improve the 3D reconstruction significantly. |
| Data exclusions | No data were excluded from the study. |
| Replication | Vif-mediated degradation assay and in vitro ubiquitination assay were repeated at least three times and all the replicates showed the similar results. All attempts at replication were successful. |
| Randomization | The protein particle images used in the final 3D reconstruction of the cryo-EM analysis were randomly split into two half datasets for the estimation of the overall resolution by Fourier shell correlation. No other methods were used that would require any randomization techniques due to the nature of the current study related to Vif-mediated degradation assay, in vitro ubiquitination assay, and single-particle cryo-EM. There is no allocation of sample groups in Vif-mediated degradation assay, in vitro ubiquitination assay, and single-particle cryo-EM and no randomization was applied. |
| Blinding | There is no allocation of sample groups in this study and no blinding was applied. All experiments were conducted in an unblinded way since the investigators were involved in the planning, execution and analyses of the current study. |

# Reporting for specific materials, systems and methods

We require information from authors about some types of materials, experimental systems and methods used in many studies. Here, indicate whether each material, system or method listed is relevant to your study. If you are not sure if a list item applies to your research, read the appropriate section before selecting a response.

### Materials & experimental systems

| n/a | Involved in the study | |
|---|---|---|
| ☐ | ☒ | Antibodies |
| ☐ | ☒ | Eukaryotic cell lines |
| ☒ | ☐ | Palaeontology and archaeology |
| ☒ | ☐ | Animals and other organisms |
| ☒ | ☐ | Clinical data |
| ☒ | ☐ | Dual use research of concern |

### Methods

| n/a | Involved in the study | |
|---|---|---|
| ☒ | ☐ | ChIP-seq |
| ☒ | ☐ | Flow cytometry |
| ☒ | ☐ | MRI-based neuroimaging |

## Antibodies

| | |
|---|---|
| Antibodies used | Monoclonal ANTI-FLAG® M2 antibody (Catalog #F3165, Sigma, 1:3,000)<br>Monoclonal ANTI-HA antibody (Catalog #H9658, Sigma, 1:3,000)<br>Monoclonal anti-tubulin antibody (Catalog #GT114, GeneTex, 1:5,000)<br>Monoclonal anti-HIV-1 Vif antibody (National Institutes of Health AIDS Reagent Program #319, 1:2,000)<br>Cy3-labelled goat-anti-mouse secondary antibody (Catalog #PA43009, GE Healthcare, 1:3,000) |
| Validation | Monoclonal ANTI-FLAG® M2 antibody: https://www.sigmaaldrich.com/US/en/product/sigma/f3165<br>Monoclonal ANTI-HA antibody: https://www.sigmaaldrich.com/US/en/product/sigma/h9658<br>Monoclonal anti-tubulin antibody [GT114]: https://www.genetex.com/Product/Detail/alpha-Tubulin-antibody-GT114/GTX628802<br>Monoclonal anti-HIV-1 Vif antibody: https://www.hivreagentprogram.org/Catalog/HRPMonoclonalAntibodies/ARP-6459.aspx<br>Cy3-labelled goat-anti-mouse secondary antibody: https://www.cytivalifesciences.com/en/us/shop/protein-analysis/blotting-and-detection/blotting-standards-and-reagents/amersham-ecl-plex-cydye-conjugated-antibodies-p-05749#productsupport |

## Eukaryotic cell lines

Policy information about cell lines and Sex and Gender in Research

| | |
|---|---|
| Cell line source(s) | 293T (or HEK293T) Cells from ATCC. |
| Authentication | No authentication procedure for the cell line was used. |
| Mycoplasma contamination | No contamination. |
| Commonly misidentified lines<br>(See ICLAC register) | No commonly misidentified cell lines were used in this study. |
